# Clear Cell Sarcoma Incidence and Survival: A Surveillance, Epidemiology, and End Results (SEER) Database Analysis

**DOI:** 10.7759/cureus.85606

**Published:** 2025-06-09

**Authors:** Harvey Y Wang, Laura M Cogua, Connor J Tupper, Peter T Silberstein

**Affiliations:** 1 College of Medicine, Creighton University School of Medicine, Phoenix, USA; 2 Department of Orthopaedic Surgery, Hofstra University/Northwell Health (Zucker) School of Medicine, Hempstead, USA; 3 Department of Oncology, Creighton University School of Medicine, Omaha, USA

**Keywords:** clear cell sarcoma, incidence, malignant melanoma, prognosis, seer database

## Abstract

Background

Clear cell sarcoma (CCS) is a rare soft tissue cancer that predominantly affects young to middle-aged adults. Current literature lacks recent and accurate estimates of patient outcomes due to the disease's low incidence and the small sample sizes in existing studies. This study aims to examine the incidence and survival of patients with CCS.

Methods

Patients from the Surveillance, Epidemiology, and End Results (SEER) database diagnosed with CCS between 2000 and 2019 were selected. Additional variables collected included age, sex, race, stage, metastases, tumor size, treatment status for surgery, radiation, and chemotherapy, median household income, and population size. Descriptive statistics, population-based incidence, chi-square tests, and Cox regression analyses were performed.

Results

A total of 268 patients were included. The population-adjusted incidence ranged from 0.012/100,000 to 0.027/100,000. The total percent change over the study period was 16.751%, and the annual percent change was 0.561%. The survival rates at one, three, and five years were 78.4%, 62.0%, and 57.1%, respectively. Cox regression results showed that Black patients (p = 0.007), tumor size greater than 4.0 cm (p = 0.033), and the presence of metastases (p = 0.040) were all associated with shorter survival.

Significance

The findings showed that CCS incidence has remained unchanged in recent years and that prognosis is poor. Black patients were found to have shorter survival durations. Contrary to prior findings, staging and tumor size were only significantly associated with survival in univariate analyses. Limitations include a small sample size and the constraints of variables available in the SEER database. Nonetheless, future research will benefit from assessing whether race is an independent risk factor for CCS survival and from exploring ways to improve prognosis.

## Introduction

Clear cell sarcoma (CCS) is a rare soft tissue malignancy that accounts for 1% of soft tissue sarcomas [1]. Most cases occur in adolescents and young adults, with an average age between 20 and 40 years, presenting as deep soft tissue masses located around tendons, aponeuroses, and other fascial structures in the extremities [2,3]. Due to its histological and clinical similarities to malignant melanoma (MM), CCS is also known as MM of soft parts [4,5].

Prognostic outcomes are poor, with average five- and ten-year survival rates of 41% and 37%, respectively [6]. Even when treated with optimal surgical resection, a high percentage of patients experience recurrence, underscoring the severity and aggressiveness of CCS [7].

Due to its low incidence and the small sample sizes in previous studies, recent and accurate estimates of CCS outcomes are lacking in the current literature. This study aims to evaluate the incidence and survival of patients with CCS using the Surveillance, Epidemiology, and End Results (SEER) database.

This article was previously presented as a meeting abstract at the AVAHO 2024 Annual Meeting on September 21, 2024.

## Materials and methods

Study design

This is a retrospective cohort study utilizing data from the SEER database, a population-based registry that includes 47.9% of all cancer diagnoses in the United States annually [8]. The database contains comprehensive demographic, clinical, and socioeconomic information on each case, making it a reliable source for assessing national oncological trends and outcomes.

Participant selection****

We identified cases of CCS diagnosed between January 1, 2000, and December 31, 2019, using the International Classification of Diseases for Oncology (ICD-O-3) histology code 9044. Inclusion criteria required at least one year of follow-up to ensure accurate calculation of one-year survival rates. Patients were excluded if the cause of death was not attributed to cancer, according to the SEER cancer-specific death categorization.

Variables and definitions

The primary outcome was cancer-specific survival, tracked for up to 60 months post-diagnosis to ensure an appropriate follow-up period. The following variables were collected:

Demographic Variables

Age (0-29, 30-49, ≥50 years), sex (male, female), and race/ethnicity (Non-Hispanic White, Non-Hispanic Black, Hispanic, Asian, American Indian/Alaska Native).

Clinical Variables

Tumor stage at diagnosis (localized, regional, distant), tumor size (0-2.0 cm, 2.1-4.0 cm, >4.0 cm), presence of metastatic sites (bone, brain, liver, lung), number of metastatic sites (none, one, two or more), and treatment modalities such as surgery, radiation, and chemotherapy (yes, no).

Socioeconomic Variables

Time from diagnosis to treatment (≤1 month, 1-2 months, >2 months), median household income in the patient’s county of residence (<$50,000; $50,000-$74,999; ≥$75,000), and county classification based on population density and proximity to urban centers (metropolitan, non-metropolitan).

Study size and bias

To maximize statistical power, all eligible cases found within the SEER database between the years 2000-2019 were included in the study. Only patients with cancer-specific mortality were included. As this was a fixed dataset, no formal sample size calculation was performed. Selection bias was minimized by including all eligible cases in the SEER database within the established timeframe.

Statistical analysis

Incidence rates were calculated using SEER*Stat (Version 8.4.3), standardized to United States census data [9]. Descriptive statistics were used to summarize cohort characteristics. Chi-square tests were used to compare one-year and five-year survival rates between surgical groups. Cox proportional hazards models were applied to determine associations between covariates and cancer-specific survival. The significance level for all analyses was set at alpha = 0.05, and analyses were conducted using SPSS Version 29 (IBM Corp., Armonk, NY, USA).

## Results

**Figure 1:**
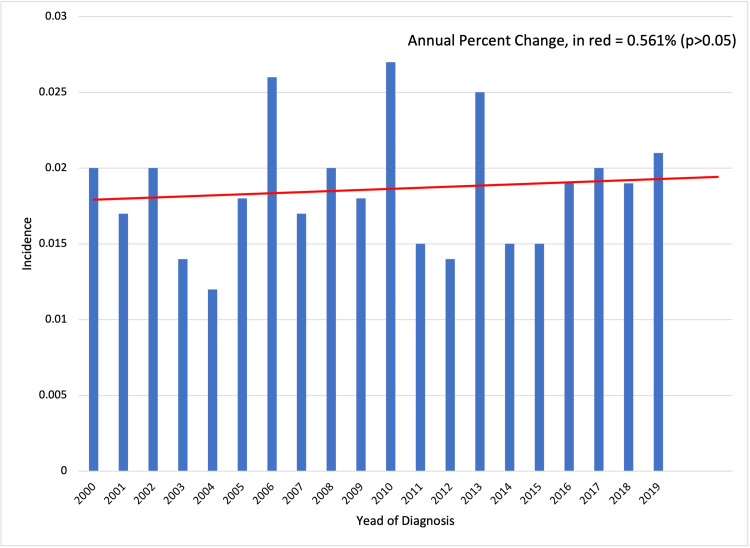
Average incidence of clear cell sarcoma between 2000 and 2020.

A total of 268 patients were included in the study. The population-adjusted incidence ranged from 0.012/100,000 in 2004 to a peak of 0.027/100,000 in 2010. The total percent change over the study period was 16.751%, and the annual percent change, which did not vary significantly over time, was 0.561% (Figure 1).

Of the included patients, 146 (54.5%) were male. A total of 148 (55.4%) patients were White, 59 (22.1%) were Hispanic, and 39 (14.6%) were Black. Regarding disease stage, 99 (46.3%) had localized staging, followed by 63 (29.4%) with regional staging and 52 (24.3%) with distant staging. A total of 92 (51.7%) had tumors greater than 4.0 cm. Surgical intervention was performed in 217 patients (81.0%). Additionally, 166 (61.9%) did not receive radiation therapy, and 209 (61.9%) did not receive chemotherapy. Among cases with known metastatic status, 116 (82.9%) had no metastasis, 15 (10.7%) had metastasis to one site, and 9 (6.4%) had metastasis to two sites. Treatment timing relative to diagnosis showed that 168 (66.9%) of patients were treated within one month of diagnosis. Complete descriptive statistics are presented in Table 1.

**Table 1:** Patient variables and their associations with one- and five-year survival following a diagnosis of clear cell sarcoma. Note: p < 0.05 indicates statistical significance.

Variable	N	One-Year Survival: Yes	One-Year Survival: No	P-value	Five-Year Survival: Yes	Five-Year Survival: No	P-value
Age	-	-	-	-	-	-	-
0-29 years	94 (35.1)	72 (34.3)	22 (37.9)	0.305	45 (38.5)	29 (33.0)	0.588
30-49 years	101 (37.7)	84 (40.0)	17 (29.3)	-	43 (36.8)	32 (36.4)	-
50+ years	73 (27.2)	54 (25.7)	19 (32.8)	-	29 (24.8)	27 (30.7)	-
Gender	-	-	-	-	-	-	-
Male	146 (54.5)	111 (52.9)	35 (60.3)	0.311	56 (47.9)	59 (67.0)	0.006
Female	122 (45.5)	99 (47.1)	23 (39.7)	-	61 (52.1)	29 (33.0)	-
Race	-	-	-	-	-	-	-
White	148 (55.4)	120 (57.4)	28 (48.3)	0.077	75 (64.7)	42 (47.7)	0.03
Black	39 (14.6)	25 (12.0)	14 (24.1)	-	9 (7.8)	19 (21.6)	-
Hispanic	59 (22.1)	45 (21.5)	14 (24.1)	-	24 (20.7)	18 (20.5)	-
Asian	18 (6.7)	17 (8.1)	1 (1.7)	-	6 (5.2)	8 (9.1)	-
American Indian/ Alaska Native	3 (1.1)	2 (1.0)	1 (1.7)	-	2 (1.7)	1 (1.1)	-
Staging	-	-	-	-	-	-	-
Localized	99 (46.3)	96 (56.8)	3 (6.7)	<0.001	60 (69.0)	14 (20.9)	<0.001
Regional	63 (29.4)	50 (29.6)	13 (28.9)	-	24 (27.6)	22 (32.8)	-
Distant	52 (24.3)	23 (13.6)	29 (64.4)	-	3 (3.4)	31 (46.3)	-
Tumor Size	-	-	-	-	-	-	-
0-2.0 cm	32 (18.0)	30 (21.4)	2 (5.3)	<0.001	18 (26.9)	4 (6.7)	<0.001
2.1-4.0 cm	54 (30.3)	49 (35.0)	5 (13.2)	-	26 (38.8)	15 (25.0)	-
4.1+ cm	92 (51.7)	61 (43.6)	31 (81.6)	-	23 (34.3)	41 (68.3)	-
Any Metastasis	-	-	-	-	-	-	-
None	116 (82.9)	101 (89.4)	15 (55.6)	<0.001	47 (100.0)	20 (62.5)	<0.001
1 site	15 (10.7)	8 (7.1)	7 (25.9)	-	0 (0.0)	7 (21.9)	-
2+ sites	9 (6.4)	4 (3.5)	5 (18.5)	-	0 (0.0)	5 (15.6)	-
Surgery Status	-	-	-	-	-	-	-
No	51 (19.0)	23 (11.0)	28 (48.3)	<0.001	10 (8.5)	25 (28.4)	<0.001
Yes	217 (81.0)	187 (89.0)	30 (51.7)	-	107 (91.5)	63 (71.6)	-
Radiation Status	-	-	-	-	-	-	-
No	166 (61.9)	130 (61.9)	36 (62.1)	0.982	78 (66.7)	49 (55.7)	0.109
Yes	102 (38.1)	80 (38.1)	22 (37.9)	-	39 (33.3)	39 (44.3)	-
Chemotherapy Status	-	-	-	-	-	-	-
No	209 (78.0)	179 (85.2)	30 (51.7)	<0.001	108 (92.3)	51 (58.0)	<0.001
Yes	59 (22.0)	31 (14.8)	28 (48.3)	-	9 (7.7)	37 (42.0)	-
Time to Treatment	-	-	-	-	-	-	-
< 1 month	168 (66.9)	142 (70.3)	26 (53.1)	0.071	87 (77.7)	47 (57.3)	0.007
1-2 months	54 (21.5)	39 (19.3)	15 (30.6)	-	16 (14.3)	26 (31.7)	-
2+ months	29 (11.6)	21 (10.4)	8 (16.3)	-	9 (8.0)	9 (11.0)	-
Median Household Income	-	-	-	-	-	-	-
Population Size	-	-	-	-	-	-	-
Metropolitan	236 (88.1)	185 (88.1)	51 (87.9)	0.973	102 (87.2)	75 (85.2)	0.687
Non-metropolitan	32 (11.9)	25 (11.9)	7 (12.1)	-	15 (12.8)	13 (14.8)	-

The one- and five-year survival rates were 78.4% and 57.1%, respectively. Chi-square analysis demonstrated that stage, tumor size, metastases, surgery status, and chemotherapy status were all significantly associated with one-year survival (p’s < 0.001). No significant relationships were found between one-year survival and age, gender, race, radiation status, time to treatment, median household income, or population size. Factors significantly associated with five-year survival included gender, race, stage, tumor size, metastases, surgical status, chemotherapy status, and time to treatment (p’s < 0.05). There were no significant associations between five-year survival and age, radiation status, median household income, or population size. Complete chi-square comparisons are presented in Table 1.

On Cox regression, Black patients were associated with decreased survival (p = 0.007, HR = 4.634, 95% CI = 1.508-14.247). Patients with tumor size over 4.0 cm were associated with decreased survival (p = 0.033, HR = 9.686, 95% CI = 1.197-78.393). Patients with metastasis to one site were also associated with decreased survival (p = 0.040, HR = 4.360, 95% CI = 1.071-17.741). No other significant findings were observed for age, gender, race (beyond the Black subgroup), surgery, radiation, chemotherapy, time to treatment, median household income, or population size. Full results are presented in Table 2.

**Table 2:** Cox regression analysis of patient outcome variables following a clear cell sarcoma diagnosis. Note: p < 0.05 indicates statistical significance.

Variable	P-value	Hazard Ratio	95% CI
Age (ref: 0-29 years)	-	-	-
30-49 years	0.278	0.620	0.262-1.469
50+ years	0.630	0.781	0.287-2.129
Gender (ref: Male)	-	-	-
Female	0.488	0.755	0.342-1.668
Stage (ref: Localized)	-	-	-
Regional	0.192	2.006	0.705-5.704
Distant	0.158	3.150	0.640-15.507
Race (Ref: White)	-	-	-
Black	0.007	4.634	1.508-14.247
Hispanic	0.120	1.925	0.843-4.396
Asian	0.188	2.642	0.621-11.236
American Indian/ Alaska Native	0.483	3.216	0.123-84.044
Tumor Size (ref: 0-2.0 cm)	-	-	-
2.1-4.0 cm	0.136	4.931	0.604-40.225
4.1+ cm	0.033	9.686	1.197-78.393
Any Metastasis (ref: None)	-	-	-
1 site	0.040	4.360	1.071-17.741
2+ sites	0.457	0.529	0.099-2.836
Surgery Status (ref: No)	-	-	-
Yes	0.098	0.384	0.124-1.191
Radiation Status (ref: No)	-	-	-
Yes	0.519	1.298	0.588-2.865
Chemotherapy Status (ref: No)	-	-	-
Yes	0.631	0.772	0.269-2.219
Time to Treatment (ref: <1 month)	-	-	-
1-2 months	0.923	1.047	0.412-2.664
2+ months	0.122	0.404	0.128-1.273
Median Household Income (ref: < $50,000)	-	-	-
$50,000-$74,999	0.666	0.708	0.148-3.399
$75,000+	0.879	0.879	0.166-4.643
Population Size (ref: Metropolitan)	-	-	-
Non-Metropolitan	0.715	0.718	0.121-4.257

## Discussion

The results of this retrospective analysis of 268 CCS patients indicate that there has been no significant increase in incidence, and the CSS remains poor, with one- and five-year survival rates at 78% and 57%, respectively. After controlling for covariates, Black race, metastases at diagnosis, and tumor size greater than 4.0 cm were associated with shorter survival.

The nonsignificant increase in CCS incidence may be partly attributed to the diagnostic challenge posed by its similarities to the more common primary MM [10]. To distinguish CCS from MM, molecular analysis is required to identify the cytogenetic hallmark translocation t(12;22)(q13;q12), which confirms a CCS diagnosis [11,12]. Consequently, CCS diagnosis is complex and necessitates molecular and cytogenetic testing [13]. Without these, CCS may be easily misdiagnosed as metastatic MM, leading to underreporting of its true incidence and prevalence.

The overall five-year prognosis for patients diagnosed with CCS remains unfavorable. Previous literature has reported five-year survival rates ranging from 41% to 63% [6,14-19], and our observed rate of 57.1% aligns with this range. The poor prognosis may be explained in part by CCS's origin from neural crest cells, which are prone to early metastasis to the lungs and via the lymphatic system [20]. Moreover, due to its characteristic chromosomal translocation, CCS shows poor responsiveness to conventional chemotherapy, making advanced cases difficult to treat [21,22]. This is reflected in our findings: 209 patients (78%) did not receive chemotherapy, and survival among those who did was only 52.5% at one year and 19.6% at five years. Future research into improved therapies or drug regimens may help enhance outcomes for advanced CCS cases.

Tumor size greater than 4.0 cm was significantly associated with poorer survival in this study. This finding is supported by earlier literature: Li et al. found tumor size over 3 cm correlated with worse outcomes compared to tumors smaller than 3 cm [14]. Similarly, Zamora et al., using data from the National Cancer Database, identified large tumor size as the sole risk factor significantly associated with lower survival in CCS patients [23,24]. Kawai et al. reported that tumors larger than 5 cm were linked with worse prognosis [25]. The impact of tumor size on survival may be due to the increased likelihood of metastasis or recurrence associated with larger tumors [26]. Larger tumors often necessitate more aggressive surgical interventions, such as wide resection with negative margins or even amputation, as opposed to the less effective intralesional excision, because of their higher risk of distant spread [2,27,28]. This may reflect their greater likelihood of invading local tissues and lymph nodes, complicating disease control.

In our investigation, the presence of metastases at diagnosis was associated with worse survival, while advanced staging only approached significance, likely due to the small sample size. However, it is well established that higher stage is a key prognostic factor for CCS. Patients diagnosed at a localized stage had a 3.2-fold lower risk of death compared to those diagnosed at a regional stage [14]. Distant staging has also been associated with reduced survival compared to earlier stages in multiple reports [15,16]. Research focused on cases with advanced staging, as well as efforts to detect tumors before metastasis, will be critical to improving CCS prognosis.

Our findings suggest that Black patients experience shorter survival compared to White patients. A comprehensive review of prior studies did not identify a known association between race and CCS prognosis. While some investigations reported a lower prevalence of CCS among Black patients compared to White patients [16,23], they did not establish a survival correlation. Our results may be partly influenced by the small number of Black patients in the sample; however, further research assessing demographic variables and their influence on CCS outcomes is warranted.

This study has several limitations. Despite using a national database, SEER only provides retrospective data, meaning that findings can indicate correlations rather than causation, as would be possible in prospective studies. Although this analysis uses the largest population dataset to date for CCS, the rarity of the disease still limits the overall sample size. Additionally, registry-based analyses often lack detailed clinical information and contextual factors that could influence patient outcomes. Real-world studies are therefore needed to validate our findings. Furthermore, patient comorbidities were not available in the SEER database, which could have provided further insight into additional risk or prognostic factors. As a result, inherent biases must be considered when interpreting and drawing conclusions from this data.

## Conclusions

The incidence of CCS has remained stable in recent years. Prognosis also continues to be poor at both one- and five-year intervals, highlighting the need for advances in treatment. Greater disease staging, tumor size >4.0 cm, and Black race were associated with decreased survival. These findings provide updated information that can inform clinical practice and management strategies for patients diagnosed with CCS. Healthcare providers can use this data to better educate patients and support informed decision-making when developing individualized treatment plans. Due to the rarity of CCS and the limited availability of specific clinical data within the SEER database, this study serves as an initial step toward identifying key prognostic factors in CCS management. Future research would benefit from a more detailed analysis of clinical and demographic variables influencing survival, as well as the development of more targeted therapies, particularly for advanced-stage disease.
